# Preparing synthetic biology for the world

**DOI:** 10.3389/fmicb.2013.00005

**Published:** 2013-01-25

**Authors:** Gerd H. G. Moe-Behrens, Rene Davis, Karmella A. Haynes

**Affiliations:** ^1^Leukippos InstituteBerlin, Germany; ^2^School of Biological and Health Systems Engineering, Arizona State UniversityTempe, AZ, USA

**Keywords:** synthetic biology, biosafety research, containment of biohazards, risk assessment

## Abstract

Synthetic Biology promises low-cost, exponentially scalable products and global health solutions in the form of self-replicating organisms, or “living devices.” As these promises are realized, proof-of-concept systems will gradually migrate from tightly regulated laboratory or industrial environments into private spaces as, for instance, probiotic health products, food, and even do-it-yourself bioengineered systems. What additional steps, if any, should be taken before releasing engineered self-replicating organisms into a broader user space? In this review, we explain how studies of genetically modified organisms lay groundwork for the future landscape of biosafety. Early in the design process, biological engineers are anticipating potential hazards and developing innovative tools to mitigate risk. Here, we survey lessons learned, ongoing efforts to engineer intrinsic biocontainment, and how different stakeholders in synthetic biology can act to accomplish best practices for biosafety.

## Beyond the lab—where synthetic organisms may appear in the future

Synthetic biologists aim to create living systems that serve immediate human needs, rather than waiting for evolution to produce a useful biological function. Reverse-engineered organisms are currently being used in closed industrial settings to produce fuels (e.g., Chromatin Inc., Ginkgo Bioworks, LS9 Inc., Solazyme, Verdezyne, and Synthetic Genomics), generate renewable chemicals of commercial value (e.g., Genencor, Genomatica Sustainable Chemicals, and Verdezyne), and reduce the cost of pharmaceutical production (e.g., Ambrx and Amyris). In these cases, preventing accidental release is straightforward. Closed industrial settings use synthetic organisms where physical containment and proper waste management can be monitored and enforced by regulatory bodies (e.g., Environmental Health and Safety groups and the Environmental Protection Agency in the U.S.). Since accidental release is still a possibility, containment mechanisms that are built into the synthetic organism could be used to enhance safe use. In contrast to closed settings, open systems (e.g., bioremediation, agriculture, and healthcare applications) distribute synthetic organisms across broader spaces in an unpredictable manner, and thus require intrinsic containment mechanisms.

Recent reports of clinical applications and anticipated uses of synthetic organisms show that the appearance of synthetic organisms in broader spaces is on the horizon. Groups in Europe have tested engineered microbes to treat human illnesses such as Crohn's disease (Braat et al., 2006) and oral inflammation (mucositis) (Caluwaerts et al., 2010). Some anticipate the use of engineered organisms in future space travel, taking engineered organisms beyond our planet (“The Initiative | Synthetic Biology,” last accessed October 21, 2012, http://syntheticbiology.arc.nasa.gov/node/1). Recent bio-fiction video projects paint intriguing pictures of engineered synthetic organisms operating in personalized contexts as consumer goods (“E. chromi,” last accessed October 22, 2012, http://vimeo.com/19759432; “Tuur van Balen—Hacking Yoghurt,” last accessed October 22, 2012, http://www.youtube.com/watch?v=Co8NOnErrPU), as living, evolving therapeutics (“Cellularity,” last accessed October 22, 2012, http://vimeo.com/10274649), and even as recreational drugs (“Compound 74,” last accessed October 22, 2012, http://www.youtube.com/watch?v=lQjF8ir4SKs). These pieces are styled to provoke the viewer with conceptual yet plausible scenarios, and to make us question where the technology may lead. Recent and speculative synthetic biology applications have catalyzed discussions of releasing synthetic organisms into the public sphere. The synthetic biology research community should respond by making visible efforts to address safe use and containment.

## Awareness, concerns, and public safety challenges

Synthetic biology is unique because of its ethos—to use design principles from nature for the rational design and construction of molecular systems with novel, reliable functions (Heinemann and Panke, 2006). Synthetic biology uses the same molecular biology practices as genetic engineering. Therefore, the techniques employed by synthetic biology do not pose any unique safety threats. Technologies that made genetic engineering a reality in the early 1970's spurred the organization of the Asilomar Conference on Recombinant DNA (rDNA) to discuss biosafety. Over the subsequent decades, concerns have waned. Now that synthetic biology has gained substantial attention and popularity, concerns about rDNA have re-emerged.

Synthetic biology has been admonished as an extreme form of genetic engineering by watch groups (“111 Organizations Call for Synthetic Biology Moratorium,” last accessed October 22, 2012, http://news.sciencemag.org/scienceinsider/2012/03/111-organizations-call-for-synth.html). Catchphrases such as “extreme genetic engineering” or “playing God,” which cast synthetic biology as a threat to human well-being, diminish the fact that the core ethos of synthetic biology, engineering (Heinemann and Panke, 2006), is a design process that aims to make human inventions reliable, predictable, and safe. Policies based on the precautionary principle could stunt the development of synthetic biology. Ironically, synthetic organisms might turn out to be the best solution for global health challenges and ecological problems such as accessible healthcare and carbon emission.

One recent study concluded that the fears that synthetic biologists are tampering with nature or “playing God” are not sufficient to establish a strong argument to restrict synthetic biology research for the sake of human well-being (Link, 2012). Along the same vein, if harnessing electricity had been restricted before the industrial revolution, mankind may never have experienced the benefits of modern electronic technologies. Still, overly optimistic promises of the benefits of synthetic biology are as unsound as than fearful perceptions. If synthetic organisms and their derivatives are to become as ubiquitous as electronic devices, then synthetic biologists must openly address the responsible and safe use of synthetic biological systems.

We can assuage fear and foster familiarity with synthetic biology through effective efforts to inform the public of the actual risks of synthetic biology research, the steps we can take to address the risks, and how this technology can be harnessed to meet society's needs. Since the 1970's, attempts have been made to address public concerns regarding the safety of genetically modified microbes (Schmidt and de Lorenzo, 2012). In 2009, the US Department of Health and Human Services released a finalized list of guidelines for identifying hazardous synthetic agents based on DNA sequence homology (“Screening Framework Guidance for Providers of Synthetic Double-Stranded DNA,” November 19, 2010, available at http://www.phe.gov/preparedness/legal/guidance/syndna/Pages/default.aspx). However, scientists have expressed doubt about the usefulness of an approach that focuses only on DNA sequences (Eisenstein, 2010). The biosafety information is enveloped in very technical language that is not accessible to non-specialists. There is little evidence that these efforts have swayed public perceptions (“Awareness and Impressions of Synthetic Biology,” September 9, 2010, available at http://www.synbioproject.org/library/publications/archive/6456/). A request for a synthetic biology moratorium released by 111 organizations including ETC Group and Friends of the Earth is an example of how the public may react when coordinated efforts toward executing containment and control strategies are not highly visible (“111 Organizations Call for Synthetic Biology Moratorium,” last accessed October 22, 2012, http://news.sciencemag.org/scienceinsider/2012/03/111-organizations-call-for-synth.html).

The Woodrow Wilson Synthetic Biology Project has recently developed a public web portal to present developments and biosafety activities in the field to non-specialists (“Synthetic Biology Project,” last accessed October 21, 2012, http://www.synbioproject.org/). In addition, the Woodrow Wilson group has proposed a framework for risk research that addresses four public safety issues (Dana et al., 2012). First, how might synthetic organisms interact with natural ones? Second, how well will they survive in receiving environments? Third, how might they evolve and adapt to fill new ecological niches? Lastly, what is the potential for gene transfer into unmodified organisms? The synthetic biology community can address these questions through designing, building, and testing synthetic systems.

## Genetic safeguards: building containment mechanisms into synthetic life

Decades of work in closed settings, such as research labs, might suggest that engineered organisms pose little threat. So far, no bio-hazardous incidents have been traced back to engineered organisms (Schmidt and de Lorenzo, 2012). Furthermore plasmids, the small circular pieces of DNA that encode engineered functions, persist poorly in host cells over time. Reduced viability in plasmid-carrying microbes compared to non-engineered parent strains has been observed (Betenbaugh et al., 1989). Nonetheless, if speculations correctly predict the future use of synthetic biology, the technology will scale to large industrial volumes, introduce large numbers of synthetic organisms into the environment for bioremediation, and be used in private spaces where dispersal and disposal are difficult to monitor. Innovative containment mechanisms will improve safety in open synthetic systems. Genetic safeguards operate within the synthetic organisms themselves to prevent escaped microbes from proliferating unchecked and to prevent the spread of engineered genetic material into unintended host cells.

### Containment through engineered auxotrophy

**Figure 1:**
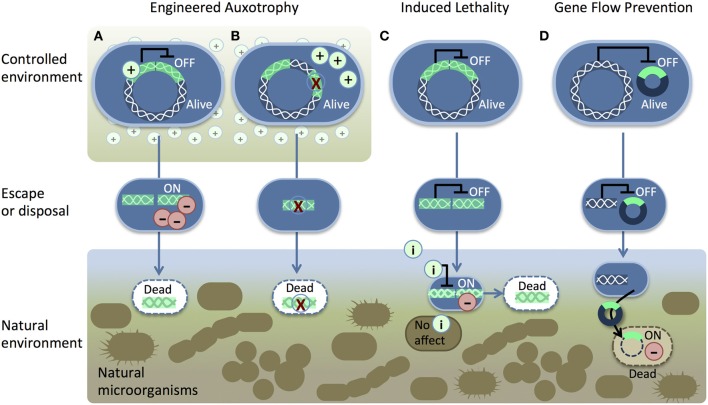
**Genetic safeguard strategies.** Recombinant DNA (bright green) is introduced into the host chromosome (white wavy lines). Two pathways for engineered auxotrophy **(A,B)** kill synthetic organisms (blue) once they lose access to a supplement (+) in a controlled environment. The supplement either **(A)** suppresses a toxic gene product (−) or **(B)** provides nutrition to compensate for a genetic deletion (red X). The induced lethality system **(C)** produces a toxic gene product (−) in response to an inducer (i) such as IPTG, sucrose, arabinose, or heat. Gene-flow prevention **(D)** is accomplished by placing a toxic gene into the recombinant DNA (dark blue/bright green circle) in an immune host. Transfer of the recombinant plasmid kills unintended host cells.

One method for biocontainment is to engineer auxotrophic organisms that are unable to synthesize an essential compound required for their survival. Once auxotrophic microbes escape the controlled environment where the compound is supplied, they rapidly die (Figures 1A,B). The first active genetic containment system, reported in 1987, used engineered auxotrophy (Molin et al., 1987). Prior to this innovation, genetically compromised bacteria were used for industrial applications. These weakened microbes may be safe to use, but this approach reduces industrial productivity and increases product cost. Molin and colleagues designed a DNA cassette that could function as a conditional suicide system in any healthy bacterial strain (Molin et al., 1987). In the absence of an artificially supplied growth supplement, the cassette produced Hok, a toxic protein that damages bacterial cell membranes (Gerdes et al., 1986) and kills the cells. Another version of this system used stochastic activation of Hok to kill a predetermined fraction of cells per unit of time (Molin et al., 1987). Stochastic activation could help to tune the level of lethality so that an optimal level of bioproduction is achieved.

A pioneering containment system for bioremediation applications was published in 1991 by Contreras et al. (1991). They designed a genetic switch to kill microbes once a mission was completed (e.g., after degrading an environmental pollutant). Cells engineered to destroy the pollutant compound benzoate remained alive in the presence of that compound. Benzoate depletion activated an artificial *xylS* gene switch, which produced Gef, a toxic protein that functions in a similar manner as Hok (Poulsen et al., 1989). Later, Jensen et al. showed that two copies of the *xylS-gef* switch improved killing of benzoate-depleted cells (Jensen et al., 1993). Further improvements were pursued by testing other toxic proteins, including streptavidin (Szafranski et al., 1997). A different gene switch has been designed for trophic containment of engineered yeast. In the absence of high glucose concentrations, the yeast express either toxic RelF or Serratia NucA DNase (Kristoffersen et al., 2000; Balan and Schenberg, 2005).

Deletions of essential genes have been used to improve the efficacy of genetic containment. Ronchel and colleagues placed a dual system in cells where the aspartate-β-semialdehyde dehydrogenase gene (*asd*) was deleted (Ronchel and Ramos, 2001). The *asd* deletion renders Pseudomonas *putida* dependent upon diaminopimelic acid, methionine, lysine, and threonine supplements. An engineered *xylS*-controlled *asd* gene was introduced into cells along with the *xylS-gef* system, so that benzoate depletion caused both production of Gef and deactivation of the growth-promoting gene *asd*. Recently, interleukin 10-secreting auxotrophic *Lactococcus lactis* (Steidler et al., 2003) has been used to treat Crohn's Disease (Braat et al., 2006). In order to prevent uncontrolled proliferation, auxotrophy was created by eliminating thymidylate synthase (*thyA*) (Steidler et al., 2003). The population of engineered bacteria fell below detection limits in the absence of thymidine and did not acquire functional thymidylate synthase from other bacteria in controlled experiments in pigs. A thorough review of biosafety practices for genetically modified *L. lactis* has been recently published (Bahey-El-Din, 2012). Engineered auxotrophy is also highly effective in eukaryotes, such as the aquatic plant *Lemna*. In *Lemna* engineered to produce therapeutic proteins and vaccines, isoleucine auxotrophy was created by using RNA interference (RNAi) to silence threonine deaminase (Nguyen et al., 2012). Engineered auxotrophy via gene knock-out or silencing can remain effective as long as gene transfer does not compensate for the mutations and as long as the nutrient that is required for survival is not available outside of the target environment.

### Active containment through induced lethality

Induced lethality (Figure 1C), or “kill switch” mechanisms have been engineered as genetic safeguards. The engineered organisms survive normally until an inducer signal (e.g., IPTG) is added. Induced lethality could be used clean up synthetic microbe spills without harming other cells in the environment. An early proof of concept switch was created by placing the toxic *hok* gene under the control of the strong and inducible *lac* promoter (Bej et al., 1988). Later, other toxic proteins that are homologous to Hok (Poulsen et al., 1989), such as RelF (Knudsen and Karlström, 1991) and Gef (Bej et al., 1992), were tested in *lac*-controlled kill switches. In microcosm studies, Knudsen and colleagues demonstrated effective IPTG-induced kill switch activation of engineered microbes in soil, seawater, and an animal model (rat intestine) (Knudsen et al., 1995). Other inducers such as heat (Ahrenholtz et al., 1994), sucrose (Recorbet et al., 1993), and arabinose (Li and Wu, 2009) have been used to activate death in engineered cells.

Recent developments in artificial cell division counters have brought us closer to timed, automatic death of synthetic cells. A set of synthetic genetic components that includes a riboregulated transcriptional cascade and a recombinase-based cascade of memory units can count up to three events (Friedland et al., 2009). These counting circuits could be designed to limit the life span of synthetic cells by linking the circuit to intracellular cell cycle-cues. Genes such as *hok*, *relF*, or *gef* could be added so that a toxic protein is produced after a certain number of cell cycles (Lu et al., 2009).

### Gene-flow barriers

In the absence of prohibitive mechanisms, plasmids are frequently transferred between microbes through conjugation (Heuer and Smalla, 2007). Furthermore, the death of an engineered organism is not necessarily accompanied by the disappearance of its rDNA. Cell-free DNA can remain functional and transferable even after exposure to harsh conditions (Lyon et al., 2010). Thus, scientists have developed systems to prevent the uptake and inheritance of engineered genetic material.

Gene-flow barriers are created by including a killer gene in the rDNA and placing the rDNA into an immune host. Immunity from the killer gene is provided by a repressor protein that blocks killer gene expression. If unintended hosts take up the engineered DNA, the lethal gene is decoupled from immunity and the new host cell dies (Figure 1D). RNA cleaving by colicin E3 reduces survival of recipient cells (Díaz et al., 1994; Munthali et al., 1996). Other systems include an additional safety measure that uses nucleases, such as *EcoRI*, to destroy DNA in recipient cells (Torres et al., 2000). Torres and colleagues created a reinforced barrier by combining colicin E3 and the *Eco*RI DNA endonuclease in a single system (Torres, 2003).

Stable integration of the rDNA may be a simpler way to effectively prevent gene-flow. For instance, integration of rDNA into an engineered microbe's chromosomes reduces transmissibility of the synthetic genetic material (Ronchel et al., 1995; Munthali et al., 1996; Panke et al., 1998; Martínez-García et al., 2011). In plants, rDNA can be inserted into chloroplast DNA instead of chromosomal DNA. Thus, the rDNA remains in stationary plant tissues more often than transmissible pollen granules (Svab and Maliga, 2007).

### Observed failures of engineered safeguards

**Figure 2:**
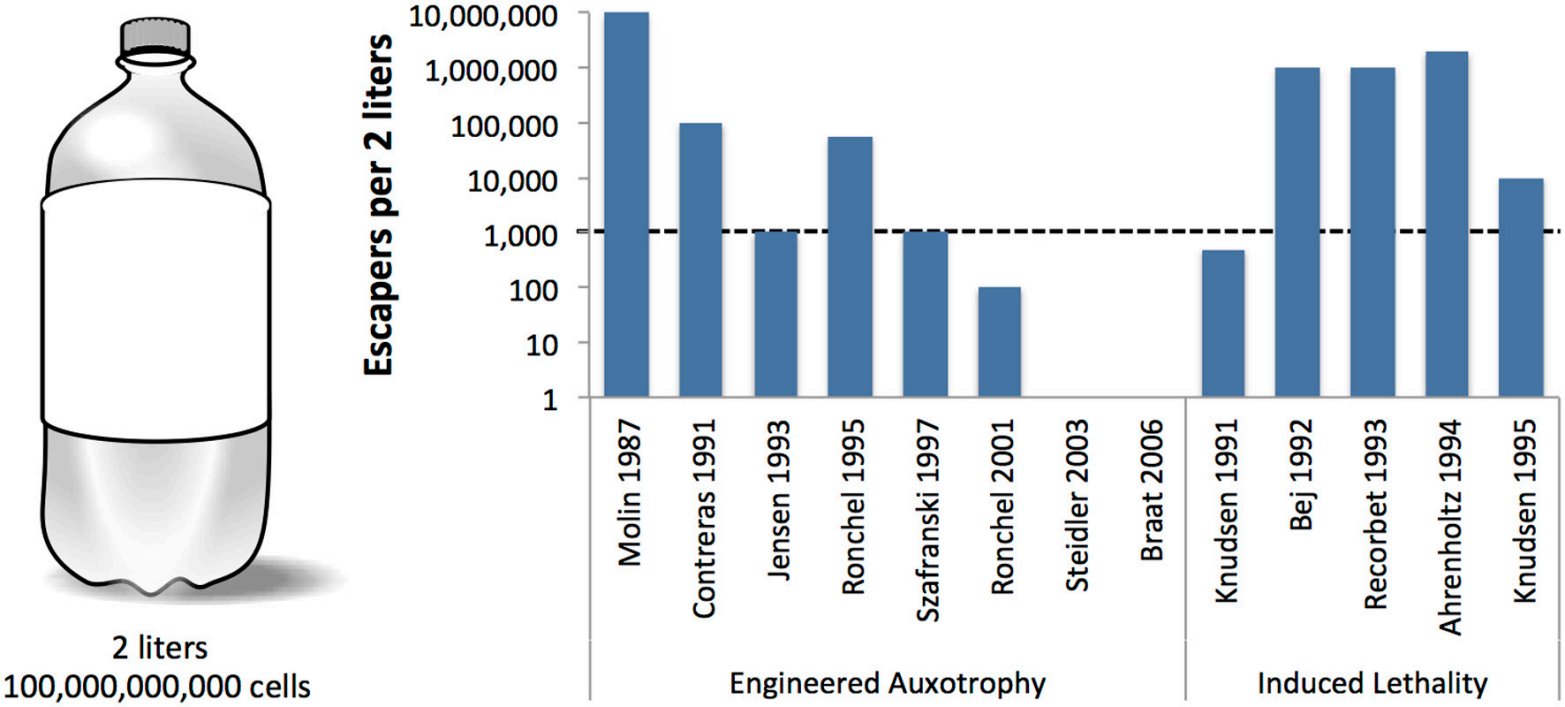
**Reported frequencies of engineered bacteria that escape various genetic safeguard systems.** A 2-liter volume is represented here as a standard soft drink container (left). Lowest reported frequencies (shown on the y-axis, log scale) were multiplied by the estimated number of cells in 2-liters at 1 × 10^8^ cells/mL, where OD600 = 0.1 [BioNumbers record ID 10985 (Milo et al., 2009)]. The dashed line indicates the maximum survival limit (1000 cells per 2 liters) recommended by the National Institutes of Health (Wilson, 1993).

Unfortunately, not all genetic safeguards are completely fail-proof. Occasionally, an engineered microbe's DNA may undergo a spontaneous mutation that destroys the genetic switch (Knudsen and Karlström, 1991) or bestows immunity against the lethal gene (Bej et al., 1988), enabling the engineered cells to propagate outside of their contained environment. Are laboratory-measured failure rates high enough to warrant serious concern? How do failure rates scale with population size? We surveyed laboratory studies of genetic safeguard systems and calculated the expected number of survivors per 2 liters, the volume of the familiar soft drink container (Figure 2). A proliferating culture of *Escherichia coli* that contains about 100 million (1 × 10^8^) cells per milliliter of culture medium [BioNumbers record ID 10985 (Milo et al., 2009)] would consist of 100 billion cells in a 2-liter volume. Table 1 shows the projected number of microbes that would survive after the activation of a genetic safeguard. These numbers are based on escape rates reported for various systems tested under laboratory conditions and in environmental microcosm models (i.e., soil, water, rat intestine). The recommended limit of engineered microbe survival or engineered DNA transmission is less than 1 cell per 10^8^ cells (Wilson, 1993), or less than 1000 cells per 2 liters, according to the National Institutes of Health. So far, only a few of the genetic safeguards meet this limit. Synthetic biologists should consider the difficulty in meeting this standard when designing genetically-contained synthetic organisms.

**Table 1:** **Lowest reported frequencies of microbes that escape engineered auxotrophy and induced lethality safeguard systems**.

**Safeguard type**	**References**	**Microbe**	**Mechanism**	**Reported survival rate**
Engineered auxotrophy	Molin et al., 1987	*B. subtilis, E. coli, P. putida*	Tryptophan *hok* switch or stochastic *hok* expression	1.00E-4
	Contreras et al., 1991	*E. coli*	*xylS-gef* switch	1.00E-6
	Jensen et al., 1993	*E. coli, P. putida*	*xylS-gef* switch (2 copies)	1.00E-8
	Ronchel et al., 1995	*P. putida*	*xylS-gef* switch (chromosome insert)	5.70E-7
	Szafranski et al., 1997	*P. putida*	*xylS-streptavidin* switch	1.00xE-8
	Kristoffersen et al., 2000	*S. cerevisiae*	*GAL1-relE* switch	Not reported
	Ronchel and Ramos, 2001	*P. putida* (in soil)	*xylS-gef* switch, gene deletion (*asd*)	1.00E-9
	Steidler et al., 2003	*L. lactis*	Gene deletion (*thyA*)	Below detection limits
	Balan and Schenberg, 2005	*S. cerevisiae* (in soil)	*ADH2*-*nucA* switch	1.00E-6
	Braat et al., 2006	*L. lactis* (in human patients)	Gene deletion (*thyA*)	Below detection limits
Induced lethality	Bej et al., 1988	*E. coli* (in soil)	IPTG-inducible *hok*	Not reported
	Knudsen and Karlström, 1991	*E. coli*	IPTG-inducible *relF*, 2 copies	5.00E-9
	Bej et al., 1992	*P. putida*	IPTG-inducible *gef*	1.00E-5
	Recorbet et al., 1993	*E. coli*	Sucrose-inducible *sacB*	1.00E-5
	Ahrenholtz et al., 1994	*E. coli*	Heat-inducible *nucA*	2.00E-5
	Knudsen et al., 1995	*E. coli* (in soil, seawater, rats)	IPTG-inducible *relF*, 2 copies	1.00E-7
	Li and Wu, 2009	*E. coli*	Arabinose-inducible *nucA*	Not reported

## Improving genetic safeguards

### Solutions for kill switch failure

Toxic gene cassettes are attractive because they enable scientists to potentially add a biocontainment mechanism to any synthetic organism. Thus, lethal genes are the most widely used feature of genetic safeguards. Unfortunately, the lethal gene is a central cause of safeguard failure. Under certain conditions, both deactivation and activation of lethal gene expression may exacerbate the failure of biocontainment. As engineered cells are passaged in the laboratory, or as they propagate in large bioreactors, broken genetic safeguards can gradually accumulate in the population. If the utility of the biocontainment mechanism is lost, then the synthetic organisms might survive in the environment after disposal or accidental release.

**Figure 3:**
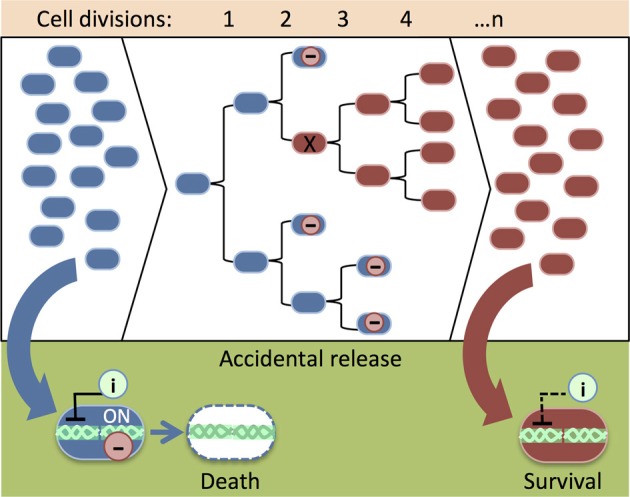
**An illustration of the accumulation of damaged genetic safeguards in a population of synthetic organisms.** When cells with intact safeguards (blue) escape physical containment (e.g., an accidental spill), an inducer (i) can be added to remove them from the environment (see Figure 1C). As the population grows, leaky expression of the lethal protein (−) reduces the viability of cells that carry functional safeguards. Mutation (X) of the lethal gene provides a growth advantage, thus cells that carry damaged safeguards (red) overwhelm the population. Cells with mutated safeguards do not respond to the cell death inducer (i). Consequently, it is difficult to remove the cells from the environment after an accidental release.

Lethal gene expression can be deactivated by spontaneous genetic mutations that arise from DNA replication error (i.e., when newly replicated DNA is not identical to its template) and DNA rearrangements (i.e., transposon mobilizations or chromosome breakage and repair) as a population of synthetic cells increases through many rounds of cell division. As a result, the population becomes non-responsive to the genetic safeguard. Knudsen and Karlström applied a classic Nobel Prize-winning approach (Luria and Delbrück, 1943) to measure the rate of spontaneous mutation of a *relF* kill switch (Knudsen and Karlström, 1991). In several trials, cells were grown for roughly 14 divisions and treated with IPTG to activate the toxic *relF* gene. Up to 49 cells survived in each experiment. Poisson distribution of survival showed that spontaneous mutations deactivated *relF* at various time points during population growth. In a population of synthetic organisms, cells carrying a mutated kill switch might gain a growth advantage and overwhelm the population (Figure 3). Experiments have demonstrated that slowing down growth by maintaining cells in a suboptimal medium and at a lowered incubation temperature prevented the accumulation of mutations that damage the lethal gene (Knudsen and Karlström, 1991). Presumably, these measures reduce the number of mutations by preventing rapid cell division.

The obvious approach for designing an effective kill switch is by expressing high levels of a lethal gene, (e.g., placing *relF* under the control of a strong promoter). What may be less obvious is that strong promoters have higher basal expression levels, which can lead to genetic safeguard failure in a microbial population. When the kill switch is in the off-state, leaky expression of the toxic gene product will lead to decreased survival of cells that have functional lethal genes. Therefore, tight repression of the lethal gene's promoter can substantially increase the survival of cells that carry a functional genetic safeguard (Knudsen and Karlström, 1991).

### Minimal genomes and orthogonal life

Scientists are developing creative new strategies that might address the shortcomings of genetic safeguards, such as the ones described in Figures 2, 3, and Tables 1, 2. Minimal genomes (Box 1) that contain only the genes that are necessary to sustain life could make many random DNA mutations lethal. Thus the likelihood of unexpected evolution and unpredictable behavior after the microbe is released into the environment would be reduced (DeWall and Cheng, 2011). Minimal synthetic chromosomes may 1 day be routinely produced by emerging technologies such as whole genome synthesis (Gibson et al., 2010) and large-scale genome editing (MAGE/CAGE; Box 1) (Isaacs et al., 2011).

**Table 2:** **Efficacy of gene-flow barriers**.

**References**	**Donor**	**Recipient**	**Gene-flow barrier**	**rDNA transfer**
Díaz et al., 1994	*E. coli*, *P. putida*	*P. putida*	Colicin E3 RNase	1.60E-8
Ronchel et al., 1995	*P. putida*	*P. putida* (in soil, water)	*Tn5* chromosome insert	Below detection limits
Munthali et al., 1996	*P. putida*	*P. putida*	Colicin E3 RNase, *Tn5* chromosome insert	1.00E-4
Torres et al., 2000	*E. coli*	*A. tumefaciens, E. coli, P. putida, R. eutropha*	EcoRI DNase	1.00E-4
Ronchel and Ramos, 2001	*P. putida*	*P. putida*	*Tn5* chromosome insert	1.00E-8
Torres, 2003	*E. coli*	*E. coli*	Colicin E3 RNase, EcoRI DNase	1.00E-8

*Lowest reported frequencies of recombinant DNA (rDNA) transfer in controlled laboratory tests are shown*.

Box 1 **Glossary of terms.**

**Minimal Genome**—a chromosome that contains only the genes that are necessary to sustain life. Pseudogenes and other non-essential DNA are removed from the chromosome.

**MAGE**—Multiplex Automated Genome Engineering.

**CAGE**—Conjugative Assembly Genome Engineering.

**Orthogonality**—(greek: orthos—“straight”, and gonia—angle) Modification of one component of a system that does not propagate side effects to other components of the system.

**Xeno**—greek: xeno—“foreign”.

Orthogonal (Box 1) life forms that use artificial genetic languages are a proposed genetic firewall that prevents the transfer of synthetic traits to natural biological systems (Schmidt and de Lorenzo, 2012). The orthogonal life form approach uses biochemical building blocks (i.e., nucleic acids and amino acids) that are incompatible with natural cells (for reviews see Liu and Schultz, 2010; Schmidt, 2010). Developments in the field of xeno (Box 1) nucleic acids (XNA) have yielded foreign genetic alphabets (Hirao et al., 2012), DNA double helix geometries, and nucleic acid backbones. Artificial bases including Ds, Px (Yamashige et al., 2012), dSICS, dMMO2 (Leconte et al., 2008; Lavergne et al., 2011), and dP, dZ (Sismour, 2004; Yang et al., 2006) preferentially bond as unnatural pairs instead of with the natural A, T, C, and G bases. Alternative DNA geometries such as expanded DNA (xDNA) and wide DNA (yDNA) (Krueger et al., 2007) are too large to fit into natural helices. Alternative nucleic acid backbones including threose (TNA), hexose (HNA), and glycol nucleic acid (GNA) (Pinheiro and Holliger, 2012) replace the natural poly-P-ribose and poly-P-deoxyribose backbones with molecular chains that cannot be replicated by natural polymerases. Recently, practical applications of orthogonal nucleic acids have been reported. XNA has been replicated in cell-free systems (e.g., PCR) (Yang et al., 2011; Betz et al., 2012; Malyshev et al., 2012), XNA has been used to express functional green fluorescent protein (GFP) (Krueger et al., 2011), and living bacteria have been evolved to use chlorouracil as a substitute for thymine (Marlière et al., 2011).

Orthogonal systems have also been engineered at the protein production level. Orthogonal amino acids have been incorporated into proteins in *E. coli* and yeast by matching an artificial transfer RNA (tRNA)/aminoacyl tRNA synthetase pair with an otherwise unused messenger RNA (mRNA) codon (the amber nonsense codon, TAG) (Wang et al., 2001; Chin, 2003) or a completely novel quadruplet codon AGGA (Anderson et al., 2004). The repertoire of functional quadruplet codons was expanded by artificially evolving a new *E. coli* ribosome (Neumann et al., 2010). Synthetic mRNA codons, tRNAs, and ribosomes make up a completely orthogonal protein translation system that may be used to create useful synthetic organisms that do not interfere with natural systems.

We are still a long way from robust orthogonal systems that can be used for practical applications. System-specific replication machinery needs to be developed to truly insulate XNA from DNA-based life forms. Current working orthogonal systems are natural-xeno hybrids. In the long term, fully orthogonal organisms could lead to a new method of engineered auxotrophy. Synthetic microbial survival would decline without a constant supply of orthogonal building blocks (XNA and xeno amino acids) from a controlled environment.

However, whether orthogonal systems can be completely insulated from the natural world is a question that is open for debate. Nucleic acids with alternative backbones (TNA, HNA, and GNA) can bond with natural DNA and RNA (Pinheiro and Holliger, 2012). This bonding could act as a toxin by interfering with DNA replication and proper gene expression. Scientists must still consider how escaped orthogonal organisms might impact natural environments.

## Risk analysis in synthetic biology

### Reporting risk-related data

Synthetic biology is at an opportune stage of development where current scientists can make risk-related analysis and data reporting a standard practice. Currently, we have methods to predict and test the environmental impact of an engineered microbe on indigenous microbial populations in soil (Corich et al., 2007; reviewed in Urgun-Demirtas et al., 2006). However, this work is limited to a few representative cases. The field needs an accepted, standard analytical method for determining the safety of a newly designed synthetic organism. We should avoid the temptation to allow representative studies and anecdotal evidence to define risk universally for every newly engineered organism.

There are many questions that must be addressed before determining appropriate risk and safety analyses. Should we expect all types of synthetic organisms to be subjected to the same risk analysis? What should standard methods for determining the safety of newly designed synthetic organism be? Until we can answer these questions, it would be prudent to include risk-related data in the original scientific report for every synthetic organism. A community-wide effort to report risk-related data would produce a wealth of information that could be parsed for meta-analyses and follow-up studies. We propose a method for reporting risk-related data (Box 2), based the Woodrow Wilson group's four focus areas for determining the safety of synthetic organisms: survival of synthetic organisms in receiving environments, gene transfer, interactions between synthetic and natural organisms, and adaptation of synthetic organisms to new ecological niches (Dana et al., 2012).

Box 2 **A hypothetical journal article section that reports risk and biosafety information for a seminal engineered genetic toggle switch (Gardner et al., 2000).**

**RISK ANALYSIS AND BIOSAFETY DATA**

**Environmental risk**:

- **Parent organism species/strain**—*E. coli* JM2.300.
- **Most likely ecological niche(s)**—None. JM2.300 is a derivative of *E. coli* K-12, a debilitated strain that does not normally colonize the human intestine and survives poorly in the environment (“*Escherichia coli* K-12 Derivatives Final Risk Assessment,” last accessed October 22, 2012, http://epa.gov/oppt/biotech/pubs/fra/fra004.htm).
- **Growth rate compared to unmodified parent strain**—Not determined.
- **Containment**—A *thi*-mutation renders JM2.300 dependent upon thiamine for growth.

**Gene transfer potential**—The toggle switch is carried on a low copy number plasmid (pBR322 ColE1 replication origin, 15–20 copies per cell). JM2.300 is an F-strain (Brenner et al., 2007). It is capable of receiving F plasmids through conjugation, and is not capable of transmitting plasmids to other microbes.

**Potential interactions**—Not determined.

**Adaptive behavior**—None identified.

We can try to predict environmental risk by considering known characteristics of the parent organism species or strain, such as the most likely ecological niches of the organism (e.g., soil, water, within a host cell). Risk that is related to synthetic modifications should also be reported. Scientists can measure synthetic versus wild type organism growth rates to determine any artificially enhanced growth (Londo et al., 2011). It is essential to determine the behavior of the synthetic organism in relevant microcosms (Bej et al., 1988; Knudsen et al., 1995; Ronchel et al., 1995; Ronchel and Ramos, 2001; Steidler et al., 2003; Balan and Schenberg, 2005), especially for engineered cells that are intended for environmental release or human and animal exposure. Reports would also include any characteristics that might aid biological containment (e.g., compromised fitness, kill switches, etc.).

The risk of gene transfer depends upon the ability of the host microbe to undergo conjugation, the viral motility of the engineered DNA, and the likelihood of plant pollination (van Elsas and Bailey, 2002; Brigulla and Wackernagel, 2010; Londo et al., 2011). The release of high-copy plasmids from dead cells might also result in gene transfer. Scientists should report rDNA transfer rates if they have collected such data (see Table 2 for examples).

In some cases, we can assume interactions between synthetic and natural organisms when the former has certain synthetic modifications. Alterations that enable synthetic cells to adhere with natural cells, invade cell membranes (Agapakis et al., 2011), or kill other cells (Russell et al., 2011) should be explicitly reported. When the synthetic organism is not explicitly designed for cellular interaction, its impact on natural cells is more difficult to predict. In this case, experiments should be done to measure the synthetic system's synergistic or toxic effect on cells it will most likely come into contact with.

The potential of synthetic organisms to adapt to new ecological niches can be reported by highlighting engineered functions that could impart adaptive behavior. For instance, synthetic systems that are engineered to survive in multiple environments could pose a containment risk. A microbe that is engineered to consume pollutants (Contreras et al., 1991) has the potential to thrive on a greater variety of nutrients than its wild-type precursor. Scientists might develop ways to make synthetic organisms more robust, perhaps by making cells less sensitive to normally toxic conditions, or by making cells invisible to the human immune system. These functions could also enhance adaptation. By predicting possible adaptations for environmental survival in advance, scientists can engineer the organisms with safety mechanisms for adequate containment.

### Using synthetic biology to detect engineered organisms in the environment

An environmental incident is an unsettling possibility that may someday call into question the safety of a synthetic organism. In this case, effective forensic tools would be critical for distinguishing synthetic from natural organisms and determining what role, if any, the synthetic organism played in the incident. Tracking techniques based on ELISA and PCR have successfully identified genetic modification markers along the agricultural pipeline, from farming (Watrud et al., 2004; Dyer et al., 2009) and harvest, through the processing (Auer, 2003). Further tracking potential is evident in a wide range of engineered markers, including detectable DNA sequences, enzymatic activity, cell surface markers could be added to synthetic organisms to aid the tracking process (Urgun-Demirtas et al., 2006). A team of college art students proposed a citizen science driven system that combined balloon cartography with PCR testing of soil samples to track the appearance of BioBrick rDNA across rural areas in India (“Searching for the Ubiquitous Genetically Engineered Machines,” last accessed October 22, 2012, http://2011.igem.org/Team:ArtScienceBangalore). This creative vision suggests how adopting genetic tagging as a standard practice today might enable surveillance methods in the future.

### Integrating risk assessment into the computer-aided design process

Software designers in the synthetic biology community are developing safeguards to help scientists prevent unintentional creation of dangerous organisms. These tools are intended to help scientists design safe synthetic systems before the system is actually built. CLOTHO is a design software tool that helps synthetic biologists construct and simulate engineered genetic devices (“Clotho,” last accessed October 22, 2012, http://www.clothocad.org/). A homology search (BLAST) against virulence factors (Chen et al., 2012) assigns risk score to modules, then alerts the user of significant overlap with potentially dangerous sequences. GenoGUARD is another open-source software tool that warns against the use of potential bioterrorism-enabling DNA agents (Adam et al., 2011; “GenoGUARD,” last accessed October 22, 2012, http://genoguard.sourceforge.net/). It uses the “best match” screening protocol method recommended by the guidelines of the US Department of Health and Human Services (“Screening Framework Guidance for Providers of Synthetic Double-Stranded DNA,” November 19, 2010, available at http://www.phe.gov/preparedness/legal/guidance/syndna/Pages/default.aspx).

How effective are these safeguards? The simple-catch approach utilized by CLOTHO is based on nucleic acid sequence homology, which is insufficient to detect multiple genetic codes that produce the same harmful protein, and does not consider emergent properties that might cause harm at the system level. Furthermore, there is no standard to rate the type and degree of potential harm. These problems can be solved with additional software features. For instance, existing protein homology algorithms could be incorporated into programs such as CLOTHO. Exploratory collaborations between computer scientists and biologists might yield new ways to predict harmfulness in higher-level properties. A more well-defined ontology for “risk” will help developers to create an extremely critical and powerful biosafety tool. This tool will prevent unintended harm at the design stage, before the synthetic organism is ever created.

### Stakeholders' roles in safe synthetic biology

In discussing best practices for biosafety, it is critical to consider the stakeholders and their roles in the growth of the synthetic biology industry. Key stakeholders include scientists, industry leaders, regulatory agencies, and the public. Exciting opportunities lie ahead, in which these various stakeholders can cooperate to shape synthetic biology. If successful, synthetic biology will stand apart from other technologies in that it is conducted in an open and ethical way. The synthetic biology industry is in its nascent stages, an ideal time to establish biosafety norms.

Many companies have sprung directly from innovations in research labs, thus scientists from those labs are also industry leaders. These industry leaders should communicate with government officials to help shape policy with effective, research-based, achievable biosafety aims instead of allowing policy to be formed from worst-case hypotheticals. However, the companies cannot objectively do their own risk assessment and Congress does not have the expertise. A recent report showed that a majority of Americans were wary of voluntary guidelines developed jointly by industry and government (“Awareness and Impressions of Synthetic Biology,” September 9, 2010, available at http://www.synbioproject.org/library/publications/archive/6456/). Thus, additional voices should also participate in the discourse around safe synthetic biology technologies. To this end, synthetic biologists, environmental microbiologists, public officials, law firm representatives, and public interest group members have recently met to discuss the best ways to address uncertainty when assessing the environmental impacts of synthetic biology (“Beyond Containment—Assessing, Testing and Demonstrating Safety on Release of Synbio Devices and Chassis,” last accessed October 22, 2012, http://www.synbioproject.org/events/archive/6635/). Similar activities in Europe, such as those organized by the SYNBIOSAFE consortium (“Synbiosafe,” last accessed October 21, 2012, http://www.synbiosafe.eu/), show that the shift toward inclusive discussion is far-reaching.

Regulatory agencies can be leveraged to monitor personal and environmental use of synthetic organisms. The US Department of Health and Human Services has released a set of guidelines to help DNA synthesis companies to only distribute safe, non-pathogenic, non-virulent nucleic acids (“Screening Framework Guidance for Providers of Synthetic Double-Stranded DNA,” November 19, 2010, available at http://www.phe.gov/preparedness/legal/guidance/syndna/Pages/default.aspx). However, more information is needed on whether novel synthetic systems might present different risks than current familiar technology. There is a gap between useful technical data in synthetic biology research reports and risk analysis. Our proposed risk-related data report (Box 2) might help to fill this gap by providing additional synthetic organism aspects, other than DNA sequences, to inform risk.

The public's power to shape biotechnology practices and industry is largely limited to antagonistic situations, such as class action lawsuits after a clinical treatment has done widespread harm. For the growing field of synthetic biology, we should ensure that the public has a chance to broaden its influence by engaging in cooperative and open dialog to help maximize the benefits of the technology as scientists seek new ways to serve society's needs.

There are many open questions regarding the safety of synthetic organisms and the repercussions of unintended harm. How will we know when an accidental release is cleaned? What will the responsible party (e.g., a for-profit company) owe to a community affected by a spill? Which governmental agency will be responsible for monitoring the company? What guidelines will the company be evaluated on? Diverse stakeholders must develop a strong culture of cooperative discourse to ensure that the technology moves forward while providing minimal harm and maximum benefit to society.

### Conflict of interest statement

The authors declare that the research was conducted in the absence of any commercial or financial relationships that could be construed as a potential conflict of interest.
